# Divergent urban–rural drivers of malaria vector ecology: a 6-year One Health longitudinal study in China

**DOI:** 10.1186/s13071-026-07507-w

**Published:** 2026-06-15

**Authors:** Enyu Xu, Guoding Zhu, Yin Wang, Zeyin Chong, Tianjiao Cheng, Jie Liang, Wei Liang, Jun Cao, Olaf Müller, Jinkou Zhao, Ruiyun Li, Guangyu Lu

**Affiliations:** 1 School of Basic Medical Sciences & School of Public Health, Faculty of Medicine, Yangzhou University, Yangzhou, 225009 People’s Republic of China; 2 National Health Commission Key Laboratory of Parasitic Disease Control and Prevention, Jiangsu Provincial Key Laboratory on Parasite and Vector Control Technology, Jiangsu Institute of Parasitic Diseases, Wuxi, 214064 People’s Republic of China; 3 School of Public Health, Nanjing Medical University, Nanjing, 211166 People’s Republic of China; 4 Yangzhou Center for Disease Control and Prevention, Yangzhou, 225002 People’s Republic of China; 5 Heidelberg Institute of Global Health, Medical School, Ruprecht-Karls-University, 69126 Heidelberg, Germany; 6 Global Fund to Fight AIDS, Tuberculosis and Malaria, 1218 Geneva, Switzerland; 7 Jiangsu Key Laboratory of Zoonosis, Yangzhou University, Yangzhou, 225009 People’s Republic of China

**Keywords:** *Anophelessinensis*, One Health, Vector ecology, Malaria elimination, Integrated surveillance, Urban–rural differences

## Abstract

**Background:**

Sustaining malaria elimination requires addressing residual transmission driven by *Anopheles sinensis* amid rapid environmental change, with a critical knowledge gap in understanding how climate, agricultural practices, and urbanization shape vector ecology. We quantified the One Health drivers of *An. sinensis* biting activity and population dynamics along urban–rural landscapes in eastern China to inform targeted control.

**Methods:**

We conducted longitudinal surveillance (2016–2021) in both rural and urban settings in eastern China. Biting rates and adult densities of *An. sinensis* were monitored using human-baited double net traps and UV-light traps. We employed a One Health concept-based analytical framework integrating generalized linear mixed models combined with distributed lag non-linear models (GLMM–DLNM) to assess lagged climate effects, regression analyses for socio-demographic, urbanization, and livestock effects, and random forests to determine variable importance. Models were validated using cross-correlation functions with the Modified Chelton method to account for temporal autocorrelation.

**Results:**

The six-year surveillance documented 49,970 *An. sinensis* specimens, confirming its dominance as the primary malaria vector. We found divergent ecological drivers between urban and rural landscapes. Temperature was the dominant predictor, with biting activity and population peaking at 27.45–30.13 °C after a 1-month lag in rural settings. Precipitation exhibited threshold effects, with significant risk peaking elevation observed between 65.57 and 99.66 mm in rural settings. In urban settings, the peak risks associated with temperature and precipitation occurred at the highest levels. In rural settings, a male-biased sex ratio increased biting rates by 12.2–14.0% (IRR = 1.122–1.140). Furthermore, crop cultivation elevated vector risk (IRR = 1.034–1.043), whereas cattle or buffalo presence provided strong zooprophylaxis (IRR = 0.667–0.780). Urbanization rate modestly increased vector density (IRR = 1.053–1.077). We identified a protective urban density paradox, where higher human population density suppressed vector populations (IRR = 0.633–0.682). Cross-correlation function analyses indicated that One Health-based models captured 37.8–90.8% of the spatiotemporal variation across sites.

**Conclusions:**

Climatic, zooprophylactic, and anthropogenic factors synergistically shape *An. sinensis* ecology. Sustaining elimination requires strategies harnessing protective urban factors and optimizing livestock management and agricultural planning in high-risk rural regions. Our One Health framework provides a replicable model for mitigating residual transmission globally.

**Supplementary Information:**

The online version contains supplementary material available at 10.1186/s13071-026-07507-w.

## Background

Despite China’s achievement of malaria-free certification in 2021, *Anopheles sinensis* remains the primary vector of *Plasmodium vivax* transmission in Asia, posing an ongoing risk of malaria re-establishment [1, 2]. This vector exhibits pronounced ecological adaptability, evidenced by its flexible host-seeking behavior, preference for breeding in rice fields, and emerging insecticide resistance [3–5]. These characteristics complicate control efforts in elimination settings [3–5]. While the influences of climate on *An. sinensis* populations are well documented [6, 7], the synergistic effects of rapid anthropogenic change on its spatiotemporal dynamics remain poorly quantified.

Agricultural intensification, livestock husbandry practices, and urban development are increasingly recognized for their collective impact on vector biting behavior and distribution [8–10]. However, existing models often fail to simultaneously quantify the synergistic effects of these multifaceted factors using high-resolution entomological surveillance data across urban–rural gradients [11]. Critical knowledge gaps persist, particularly regarding the divergence in indoor or outdoor biting behavior between microhabitats, the interaction between climatic and anthropogenic drivers (e.g., the synergies between temperature and livestock density), and the field validation of predictive models across ecological gradients. These limitations impede the development of targeted, concerted, multisectoral strategies essential to maintaining elimination status.

The persistence of *An. sinensis* as a competent malaria vector underscores the challenges of residual transmission in post-elimination settings, where conventional control strategies may prove inadequate without addressing the full spectrum of ecological complexity [12]. A One Health approach, integrating human, animal, and environmental health data, provides a framework sufficient to untangle these complex interactions [13, 14]. For instance, proximity to livestock can amplify or mitigate human exposure depending on shifts in host availability and biting preferences, while land-use changes may fragment breeding habitats or create novel larval sites [15, 16]. However, operationalizing the One Health framework in long-term empirical studies of vector ecology remains rare, creating a substantial evidence gap that impedes the adaptation of control programs to ongoing anthropogenic change.

**Fig. 1:**
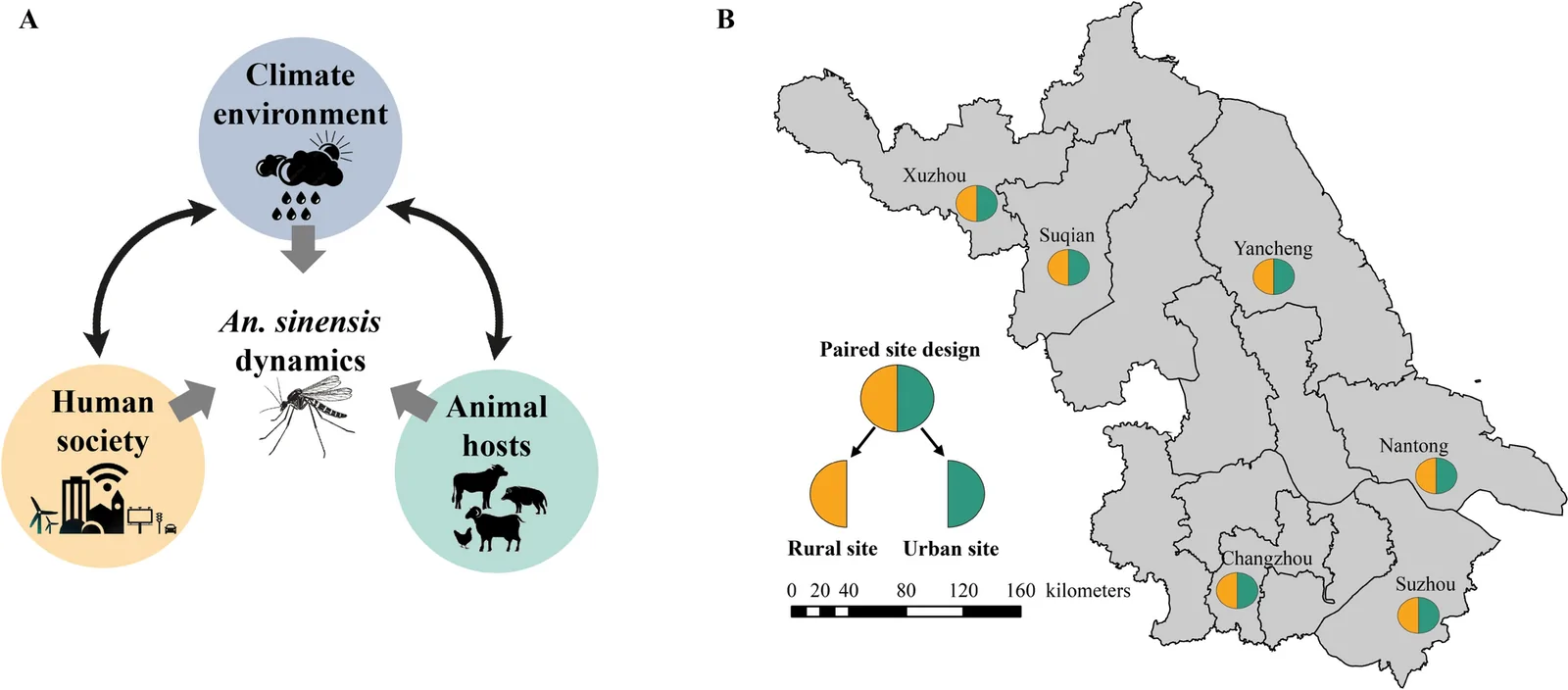
Conceptual framework and study location. **A** One Health conceptual model of the hypothesized drivers of *An. sinensis* population dynamics. We propose that mosquito ecology is governed by the complex, interactive effects of climatic, anthropogenic, and zoogenic factors, represented by bidirectional arrows. **B** Spatial distribution of the surveillance sites in Jiangsu Province. The inset illustrates the paired urban–rural sentinel site design established within each city.

To address this, we analyzed six years of longitudinal surveillance data (2016–2021) in eastern China using a One Health framework. Our study aims to (1) investigate the spatiotemporal patterns of *An. sinensis* biting rates and population dynamics across urban–rural habitat gradients; (2) quantify integrated effects of climatic (temperature/precipitation lags), zoogenic (livestock density), and anthropogenic (urbanization, sanitation infrastructure) factors through robust statistical modeling; and (3) establish evidence-based thresholds to guide targeted interventions (Fig. 1A). By bridging entomological surveillance with climate science, agricultural practices, and urban planning metrics, this study aims to provide a practical template for adaptive vector management in elimination settings.

## Methods

### Study area

This study was conducted in Jiangsu Province, China (30°45'–35°08'N, 116°21'–121°56'E), a coastal region spanning 107,200 km^2^ with a population of 85 million (26% of whom reside in rural areas) [17, 18]. The province’s diverse landscape, characterized by intensive agricultural areas and rapidly urbanizing zones, provides an ideal setting for examining vector ecology across different ecological gradients. Its transitional monsoon climate creates optimal breeding conditions for *Anopheles sinensis*, with summer (June–August) temperatures averaging 25.9 °C and rainfall exceeding 250 mm [17, 19, 20].

### Entomological surveillance

#### Data collection

Sentinel entomological surveillance was conducted from 2016 to 2021 under the China CDC’s National Mosquito-borne Disease Surveillance Program. Surveys were performed twice monthly during peak transmission seasons (June–October, extended to May–October after 2020). Six strategically selected sentinel sites were established, each containing paired rural and urban monitoring locations (Fig. 1 B and Additional file 1: Table S1) to monitor *An. sinensis* density and biting rates, thereby enabling comparisons of vector habitats [21, 22].

#### Entomological measurement

Human biting rates were assessed using human-baited double net traps (HDN) deployed indoors (within 5 m of dwellings) and outdoors (along rice paddy perimeters) from 19:00 to 24:00. The trapping approach utilized protected volunteers as bait within a nested net configuration, with an inner net measuring 120 × 120 × 200 cm and an outer net measuring 180 × 180 × 150 cm [23]. Biting rates, expressed as bites per trap per hour (b/p/h), were calculated as $HBR = \frac{M}{P\times H}$, where M is the number of mosquitoes captured, and P and H are the number of people and hours, respectively [24].

Complementary adult mosquito density data were collected using UV-light traps operated from 19:00 to 07:00 for three consecutive nights each month across all study habitats (rural dwellings, urban residences, rice fields, and urban parks) [25]. Adult density (mosquitoes per trap per hour, m/t/h) was calculated as follows: $D = \frac{M}{T\times H}$, where T is the number of traps [26]. This dual-method approach provided concurrent measures of human exposure risk and overall vector abundance.

#### Mosquito identification

All captured specimens were morphologically identified using Olympus CX31 microscopes. *An. sinensis* was confirmed based on diagnostic characteristics, including dark-scaled palps and the absence of humeral pale spots [27]. For specimens with ambiguous morphological features, molecular confirmation was conducted by PCR amplification of the mitochondrial cytochrome c oxidase subunit I (*COI*) barcode using LCO1490/HCO2198 primers [28]. This rigorous identification protocol ensured the classification of species throughout the study.

### One Health variable selection and data sources

We adopted a One Health analytical framework integrating climatic, anthropogenic, and zoogenic determinants of *Anopheles sinensis* ecology for the 6 sentinel sites. Climatic variables included monthly mean temperature (°C), precipitation (mm), and vapor pressure (hPa), all obtained from the Climatic Research Unit (CRU) datasets [29]. These meteorological parameters were selected based on their well-documented effects on mosquito life cycles and the availability of breeding habitats [30–32]. Anthropogenic factors comprised demographic measures (sex ratio and population density), economic indicators (rural and urban per capita disposable income), land-use matrices (crop area and urbanization rate), and infrastructure variables (road area and sewage treatment rate), all sourced from Jiangsu Province statistical yearbooks [33–38]. Zoogenic factors consisted of livestock data (cattle/buffalo, sheep/goats, and hogs) obtained from the Jiangsu Provincial Department of Agriculture records and the provincial statistical yearbooks and the statistical yearbooks of each city [33–39]. This comprehensive variable selection enabled an examination of the complex interplay between ecological and anthropogenic factors shaping *An. sinensis* dynamics across diverse landscapes. All demographic and environmental variables were treated as continuous, and the complete list of variables with units and sources is provided in Additional file 1: Table S2 and illustrated in Fig. 1B.

### Statistical analysis

#### Data preparation

All independent variables were normalized to ensure comparability across scales. Multicollinearity among predictors was assessed using variance inflation factors (VIFs), and variables with VIFs > 10 were excluded from the models [40]. The variance-to-mean ratios for all outcome variables (biting rates and density measures) were greater than 1, which supported the use of negative binomial regression or related models incorporating a negative binomial structure [41–43].

#### Control for repeated measures

Furthermore, we incorporated monitoring sites as a random effect into generalized linear mixed models (GLMMs) with a negative binomial distribution, using the “glmmTMB” package in R, to capture site-specific microenvironmental variation and account for non-independence in the data [44]. Combined with the natural cubic spline function of time (YearMonth, 4 df) for temporal control, this method ensured robust and unbiased estimates of variable effects and their significance. Model goodness-of-fit and explanatory power were evaluated using marginal and conditional R^2^ for generalized linear mixed models, along with residual diagnostics; the Akaike Information Criterion (AIC) was used to compare competing models [45–47].

#### Exposure-lag-response modeling

To capture delayed climatic effects, we implemented GLMMs with a negative binomial distribution and distributed lag non-linear models (GLMM-DLNM). As a sensitivity analysis, we also fit a Gamma-DLNM model. The model incorporated temperature, precipitation, and vapor pressure using natural cubic splines (3 degrees of freedom) with a 1-month lag period to align with *An. sinensis* larval development cycles [3]. Exposure-lag-response associations illustrating the impact of climate conditions at various time lags on vector populations were visualized using cross-basis matrices from R’s dlnm package [48].

#### Multivariable regression

Separate GLMM-DLNM with a negative binomial distribution was fitted for rural and urban sites, respectively, to account for the overdispersion commonly observed in mosquito vector data (e.g., human biting rate and mosquito density). For each climatic variable, incidence rate ratios (IRRs) were calculated as the ratio of the predicted incidence rate at a given exposure level to that at its reference value, defined as the mean of the respective variable [49]. For non-climatic covariates, IRRs were estimated as the relative rate change per one-unit increase in each variable, assuming linear associations. The model specification was

$$\log \left( {E\left[ {Y_{1} } \right]} \right) = \beta_{0} + ns\left( {time,df} \right) + cb\left( {C\lim ate,lag} \right) + \sum_{k} \beta_{k,t} + \gamma_{site\_id} ,$$

Where ${E[Y}_{t}]$ represents the expected biting rate or mosquito density at time t, ${\beta}_{0}$ is the intercept, ns(time) controls for long-term trends and seasonal fluctuations using natural splines, and cb(Climate,lag) captures the distributed lag effects of climate variables. The reference value for climate variables was defined as the mean value of the observed distribution. $\sum_{k}{\beta}_{k}{X}_{k,t}$ is the combined effect of anthropogenic and zoogenic covariates. *γ*_*site_id*_ was included as a random effect in the GLMM-DLNM to control for the non-independence of repeated observations from fixed monitoring sites and address potential pseudo-replication.

#### Variable importance and model validation

We implemented random forest algorithms to evaluate the relative importance of predictors, quantified as the percentage increase in mean squared error (%IncMSE) following permutation. To complement the parametric GLMM-DLNM analysis, a random forest model was applied to assess variable importance within a flexible, non-parametric framework capable of capturing non-linear relationships and interactions that may not be fully captured by the regression model. The random forest was implemented with ntree = 500, mtry = 3, and nPerm = 99 for permutation-based variable importance. Model performance was assessed using adjusted R^2^ [50]. Agreement between model-predicted values and observed bimonthly field measurements was assessed using cross-correlation functions (CCF) [51]. Correlations were evaluated to quantify the temporal synchronous agreement between predicted and observed time series. To account for temporal autocorrelation in the data, p-values were adjusted using the Modified Chelton method, which corrects for reduced effective sample size [51].

#### Software

All statistical analyses were conducted using R v4.3.1. Spatial visualizations were created in ArcGIS 10.7.

## Results

### Overall trends and the emergence of urban–rural divergence

**Fig. 2:**
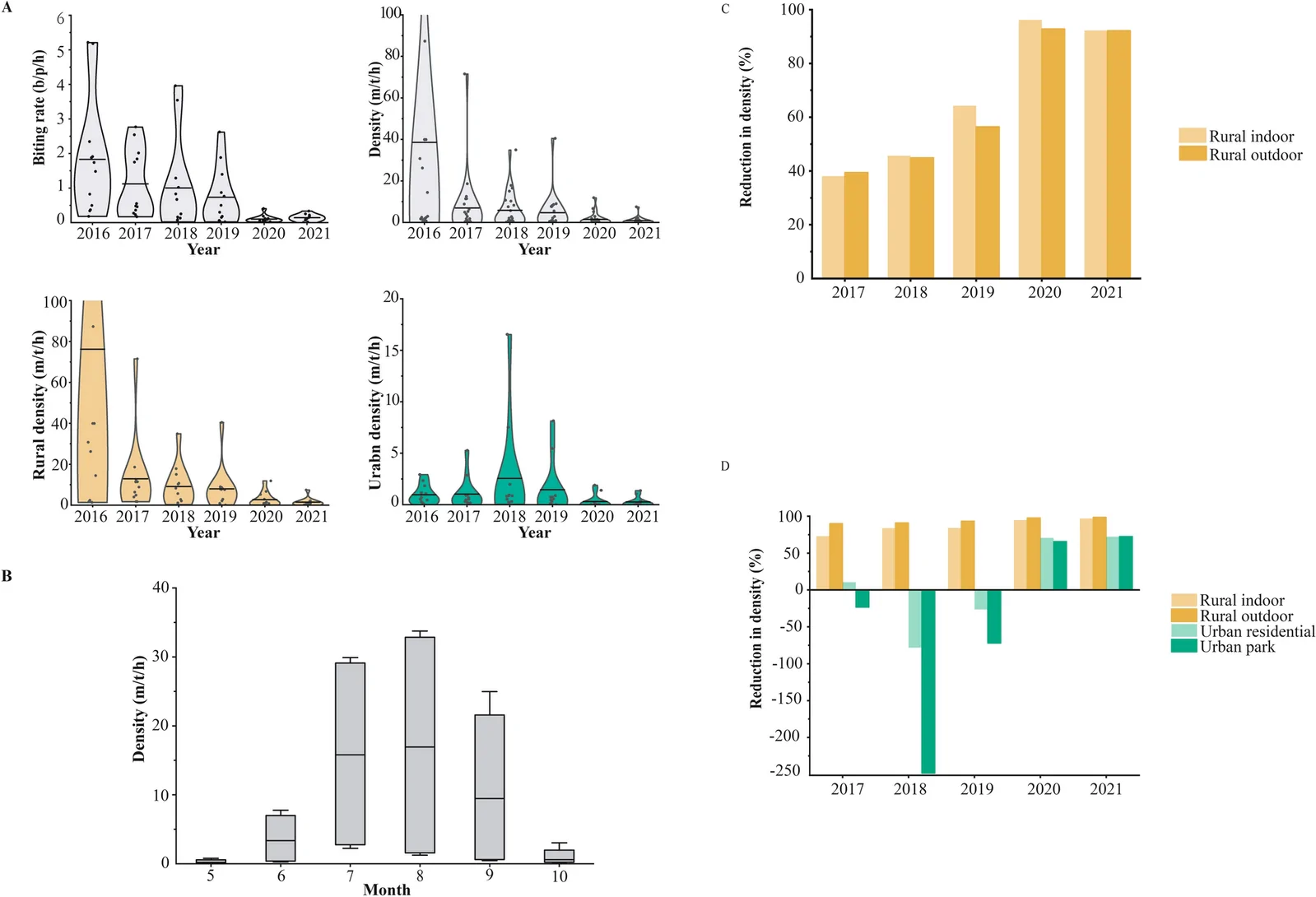
Overall trends and emergence of urban–rural divergence in *An. sinensis* populations. **A** Annual biting rate/density across habitats. **B** Seasonality of adult density over years. **C**, **D** Proportion of density reduction in different habitats

Over the six-year surveillance period, we collected 49,970 *An. sinensis* mosquitoes (4,083 by HDN and 45,887 by light traps), confirming their continuing status as the primary malaria vector in Jiangsu Province. Spatial analysis revealed fundamental divergence between urban and rural landscapes, while temporal trends showed substantial declines in mosquito density across all settings. The most pronounced temporal decline was observed in rural outdoor environments, where mosquito density decreased by 98.8% over the study period, from 88.8 to 1.1 m/t/h. In contrast, urban parks exhibited interannual variations, peaking at 3.4 m/t/h in 2018 before declining to 0.3 m/t/h by 2021 (Fig. 2A and 2C). We observed significant overall temporal declines in both biting rates and adult mosquito densities. However, consistent seasonal peaks occurred each July–August, indicating persistent vector activity during the optimal transmission months (Fig. 2B, Additional file 1: Fig. S1).

### Distinct driver profiles for rural and urban environments

Our One Health analytical approach revealed that urban and rural environments are governed by distinct sets of predictive factors. In the rural models, water vapor (VIF = 19.428), population density (VIF = 13.948), and rural per capita disposable income (VIF = 10.440) were excluded. In the urban models, water vapor (VIF = 14.842) and urban per capita disposable income (VIF = 13.098) were excluded. After removing collinear variables, models for rural sites incorporated a comprehensive suite of climatic (temperature and precipitation), human society (socioeconomic: male-biased sex ratio, crop cultivated area; urbanization: urbanization rate, road area, sewage treatment rate), and animal hosts (cattle/buffaloes, sheep/goats, and hogs) variables. Conversely, models for urban sites were driven primarily by climatic (temperature and precipitation) and human society factors (male-biased sex ratio, population density, urbanization rate, road area, and sewage treatment rate), with no significant influence from livestock and agriculture. This variable screening process itself provides quantitative evidence of a fundamental ecological shift between landscapes. The detailed characteristics of climatic, human society (socioeconomic and urbanized), and animal host variables are summarized in Additional file 1: Table S3.

### Climate responses are shaped by habitat contexts

**Fig. 3:**
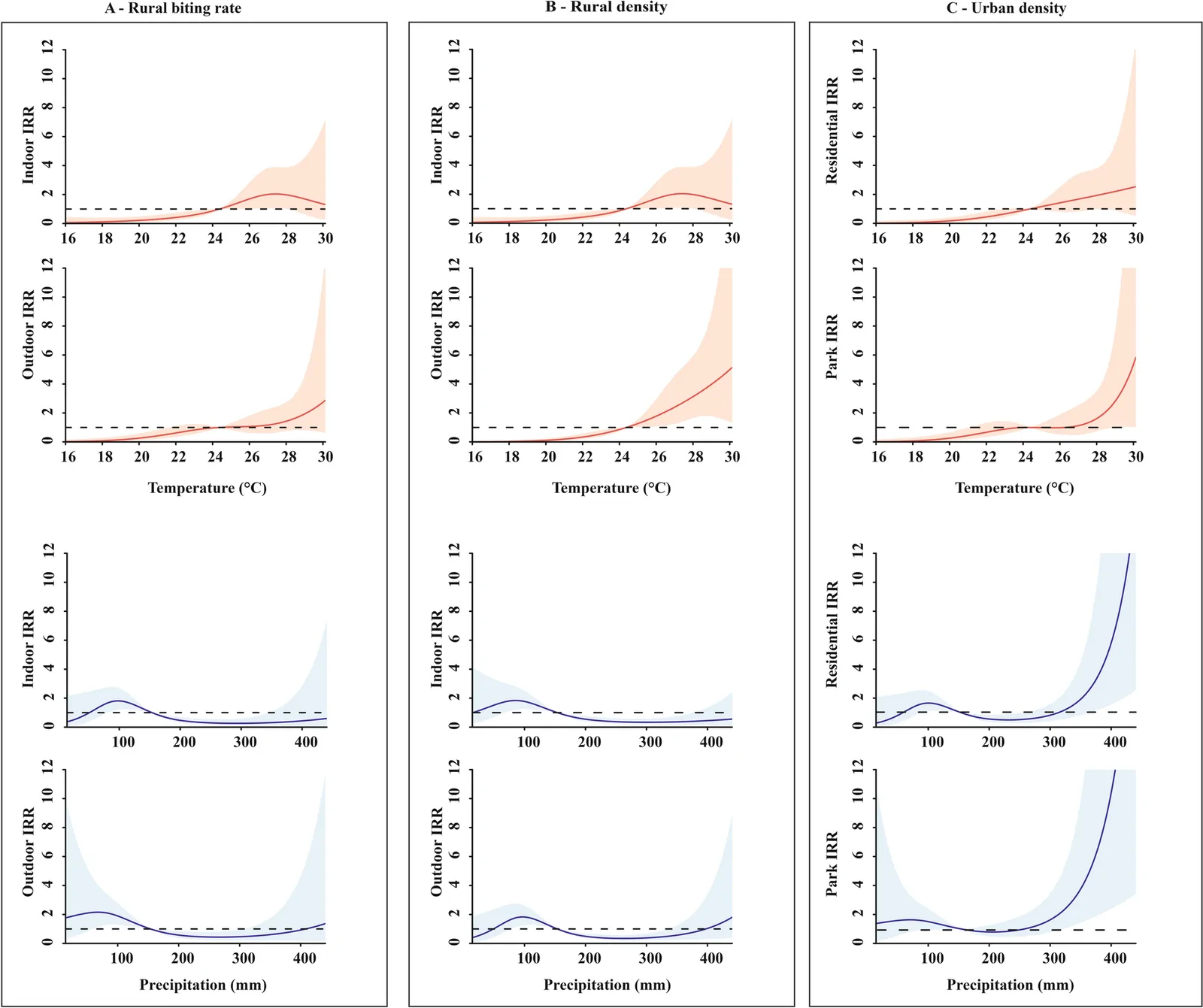
Distinct exposure-lag-response relationships of climate drivers between rural and urban habitats. Climatic drivers on **A** biting rate and **B** adult density in rural indoor and outdoor environments. **C** Attenuated and shifted climatic effects on adult density in urban residential and park environments

Temperature and precipitation exhibited distinct, non-linear relationships with *An. sinensis* dynamics, and the nature of these relationships depended on habitats (Fig. 3).

#### Rural setting

In rural habitats, climate responses were strong and clear (Fig. 3A, B). For indoor biting, risk showed a unimodal response to temperature, peaking at 27.5 °C (IRR = 2.039, 95% CI 1.068–3.895) within an optimal range of 24.5–28.1 °C. Precipitation exhibited threshold effects, with maximum risk at 99.7 mm (IRR = 1.810, 95% CI 1.223–2.680) and elevated risk between 80.3 and 152.6 mm. In outdoor environments, biting risk showed reduced risk below 21.6 °C but no statistically significant peak temperature, together with elevated risk between 58.4 and 152.2 mm of rainfall. Rural adult density showed similar climate dependencies, peaking at 27.6–30.1 °C (IRR ≈ 4.217–5.142) and 86.9–95.4 mm of rainfall (IRR ≈ 1.830–1.840) (Additional file 1: Table S4).

#### Urban setting

Urban habitats displayed attenuated and shifted climate-driven patterns (Fig. 3C). Residential areas showed a significant peak at 28.3 °C (IRR = 2.009, 95% CI 1.002–4.026), and precipitation effects were only significant at high levels (88.8–152.2 mm and > 364.8 mm). Urban parks exhibited a biting risk peak at 30.1 °C (IRR = 5.840, 95% CI 1.011–33.741) but required substantially heavier rainfall (> 320.4 mm) to impact mosquito populations, which suggests that urban infrastructure buffers typical climate effects (Additional file 1: Table S4).

### Contrasting effects of anthropogenic and zoogenic factors across urban–rural gradient

Our analysis revealed a fundamental divergence in how anthropogenic and zoogenic factors influence *An. sinensis* across urban–rural landscapes (Table 1).

**Table 1:** Key drivers of *An. sinensis* biting rates and density across habitats

Variable	Unit	Rural IRR (95%CI)		Urban IRR (95%CI)	
		Indoor	Outdoor	Residential	Parks
Adult density					
Male-biased sex ratio	1 male per 100 females	1.008(0.905–1.123)	1.130(0.998–1.279)	0.890(0.810–0.978)*	0.849(0.761–0.947)**
Population density	100 people/km^2^	–	–	0.682(0.535–0.870)**	0.633(0.465–0.861)**
Crop cultivated area	10 thousand hectares	1.028(0.968–1.093)	1.043(1.015–1.072)**	–	–
Urbanization rate	1%	1.053(1.012–1.096)*	1.026(0.988–1.066)	1.077(1.034–1.121)**	1.068(1.024–1.113)**
Sewage treatment rate	1%	0.955(0.863–1.057)	0.963(0.879–1.054)	0.929(0.838–1.029)	0.929(0.829–1.041)
Road area	1 million m^2^	0.981(0.931–1.033)	1.012(0.986–1.038)	0.994(0.973–1.015)	0.999(0.974–1.025)
Cattle/buffaloes	10,000 heads	1.060(0.786–1.429)	0.840(0.655–1.079)	–	–
Sheep/goats	10,000 heads	0.962(0.941–0.983)**	0.986(0.968–1.004)	–	–
Hogs	10,000 heads	0.998(0.994–1.002)	0.998(0.995–1.002)	–	–
Biting rate					
Male-biased sex ratio	1 male per 100 females	1.140(1.050–1.238)**	1.122(1.018–1.236)*	–	–
Crop cultivated area	10 thousand hectares	1.042(1.027–1.056)**	1.034(1.014–1.055)**	–	–
Urbanization rate	1%	0.984(0.947–1.022)	1.031(0.993–1.070)	–	–
Sewage treatment rate	1%	0.920(0.826–1.025)	0.922(0.817–1.041)	–	–
Road area	1 million m^2^	0.997(0.989–1.006)	1.006(0.991–1.021)	–	–
Cattle/buffaloes	10,000 heads	0.667(0.575–0.775)**	0.780(0.623–0.975)*	–	–
Sheep/goats	10,000 heads	0.987(0.983–0.992)**	0.994(0.985–1.004)	–	–
Hogs	10,000 heads	1(0.996–1.004)	0.998(0.995–1.002)	–	–

Incidence rate ratios (95% CIs) are presented to show the associations between anthropogenic and zoogenic variables and vector biting rates and density. Statistically significant (*p* < 0.05) variables are marked by *, and those with *p* < 0.01 are marked by **

#### Rural setting

In rural settings, human demographics and agricultural land use were significant risk factors. A male-biased sex ratio consistently increased indoor biting risk (IRR = 1.140, 95% CI 1.050–1.238) and outdoor biting rate (IRR = 1.122, 95% CI 1.018–1.236). Crop cultivated area was also positively associated with increased vector activity (indoor biting rate: IRR = 1.042, 95% CI 1.027–1.056; outdoor biting rate: IRR = 1.034, 95% CI 1.014–1.055; outdoor density: IRR = 1.043, 95% CI 1.015–1.072). Urban expansion and elevated urbanization rates were associated with an increasing trend in mosquito density (IRR = 1.053, 95% CI 1.012–1.096). Conversely, livestock presence, particularly cattle/buffalo, demonstrated a strong zooprophylactic effect. The most pronounced indoor effects were associated with a 33.3% reduction in indoor biting risk (IRR = 0.667, 95% CI 0.575–0.775) and a 22.0% reduction in outdoor biting risk (IRR = 0.780, 95% CI 0.623–0.975).

Marginal R^2^ values, representing the variance explained by fixed effects including temperature, precipitation, and other meteorological and socioeconomic covariates, ranged from 0.454 to 0.820. Conditional R^2^ values, accounting for additional variation explained by site-level random intercepts, ranged from 0.706 to 0.951 (Table 2).

**Table 2:** Fit statistics for the *An. sinensis* GLMM-DLNM models

Model	Marginal R^2^	Conditional R^2^	AIC
Rural indoor biting rate	0.820	0.820	625.2
Rural outdoor biting rate	0.608	0.706	777.3
Rural indoor density	0.454	0.951	1800.7
Rural outdoor density	0.670	0.867	1797.2
Urban residential density	0.718	0.842	639.4
Urban park density	0.709	0.864	714.9

#### Urban setting

Urban environments displayed fundamentally different ecological relationships compared to rural areas. Higher population density suppressed mosquito density in both residential areas (IRR = 0.682, 95% CI 0.535–0.870) and parks (IRR = 0.633, 95% CI 0.465–0.861). Additionally, a male-biased sex ratio was associated with reduced vector density in these urban habitats (IRR≈0.849–0.890). Similarly, urbanization rates were associated with an increasing trend in urban mosquito density (residential areas: IRR = 1.077, 95% CI 1.034–1.121; parks: IRR = 1.068, 95% CI 1.024–1.113).

Marginal R^2^ values, representing the variance explained by fixed effects including temperature, meteorological factors, and socioeconomic covariates, ranged from 0.709 to 0.718. Conditional R^2^ values, accounting for additional variation explained by site-level random intercepts, ranged from 0.842 to 0.864 (Table 2).

Moreover, we provided the results of the Gamma-DLNM model in Additional file 1: Tables S5-S6 and Figure S2. The Gamma-DLNM model produced effect estimates and significance levels similar to those from the primary GLMM-DLNM model.

### Variable importance and model validation

**Fig. 4:**
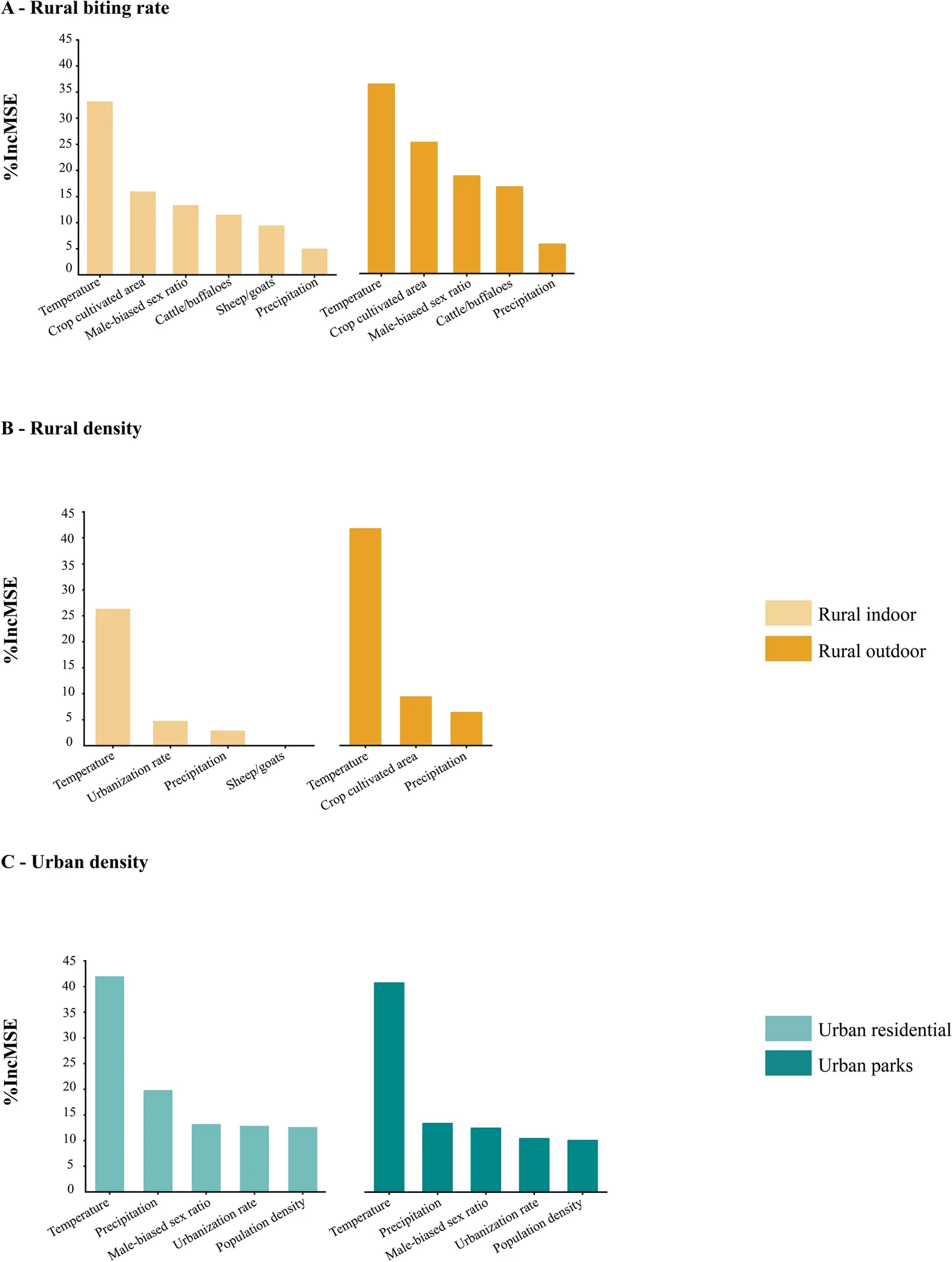
Context dependence of driver importance for An. sinensis dynamics. Predictor importance is quantified by the percentage increase in mean squared error (%IncMSE) upon permutation in random forest models. Importance rankings for **A** biting rate in rural habitats, **B** adult density in rural indoor versus outdoor habitats, and **C** adult density in urban residential areas versus urban parks

Random forest analysis identified temperature as the dominant predictor across all habitats (Fig. 4). Secondary drivers exhibited setting-specific patterns, validating the divergent ecology: rural vector dynamics were most sensitive to crop cultivated area and urbanization rate, whereas urban patterns were primarily driven by precipitation (Fig. 4 and Additional file 1: Table S7).

The integrated GLMM-DLNM framework exhibited moderate to strong model fit across diverse ecological settings. Our One Health-based models captured 37.8–90.8% of spatiotemporal variation among sites, indicating high consistency with observed values. The corresponding proportions of explained variation across different outcomes and study sites are summarized in Additional file 1: Table S8.

## Discussion

Our six-year longitudinal study provides the first comprehensive evidence that urbanization fundamentally reshapes the ecology of *An. sinensis*, creating a landscape where transmission risk is attenuated by human population structure and urban infrastructure. In contrast, rural risk remains driven by agriculture and climate. This divergence underscores a critical epidemiological shift that challenges conventional vector control and broader public health strategies aimed at sustaining malaria elimination. As other regions undergo similar rapid urbanization, our findings offer a replicable framework for understanding and managing vector-borne diseases in rapidly transitioning landscapes.

The substantial (89–98.8%) decline in rural *An. sinensis* density provides compelling evidence for the effectiveness of China's integrated vector management program, particularly the scaled-up implementation of insecticide-treated nets (ITNs) and indoor residual spraying (IRS) [52, 53]. Crucially, our results demonstrate that the protective effect of urban infrastructure is a major, previously underrecognized component of this success. However, the persistent seasonal peaks in July–August highlight the fragility of these achievements in the face of residual malaria transmission. The urban density paradox, where higher population density correlated with reduced vector populations, can be attributed to Jiangsu's advanced environmental management, including > 95% sewage treatment coverage, impermeable surfaces, near-universal paved roads, comprehensive waste collection systems, and targeted vector control in high-density zones [54–58]. This suggests that well-planned urbanization in itself can serve as a powerful public health intervention.

Our analysis confirms temperature as the principal climatic driver but reveals that its influence is heavily modified by landscape type. The unimodal response of biting rates and adult mosquito density, with a consistent one-month lag, corresponds precisely to larval development cycles and provides a biologically meaningful framework for predicting seasonal outbreaks [3]. The key novel finding is the profound attenuation of these climate-driven effects in urban settings. While rural vector dynamics were highly sensitive to moderate rainfall (91.9–109.1 mm), urban parks required extreme precipitation events (> 324 mm) to significantly impact mosquito populations. This suggests that the built environment buffers natural climatic fluctuations, likely owing to microclimate modifications by heat-mitigating infrastructure and efficient drainage systems [59, 60].

Our findings reveal two contrasting patterns: urbanization rates correlate positively with both rural and urban mosquito densities, whereas urban population density correlates negatively with urban mosquito density. These patterns can be explained by a key feature of China’s urbanization: urban land expansion has consistently outpaced population growth [77]. In rural areas, rising urbanization drives population outflow, leading to idle housing, neglected land use, and weakened vector control, all of which enhance mosquito breeding sites [62–64]. In urban settings, the imbalance between land and population expansion creates transition zones with poor infrastructure (e.g., urban villages), which provide abundant breeding habitats and increase mosquito density [65]. Conversely, higher urban population density—reflecting internal agglomeration rather than macro-level expansion—reduces mosquito density through better infrastructure, frequent human disturbance, and more effective control measures, thereby offsetting the risks associated with land expansion. This finding provides a basis for targeted mosquito control [61, 66, 67].

Beyond infrastructure, we found robust evidence for the zooprophylactic effects of livestock, particularly cattle/buffalo. The strong protective association (IRR = 0.667–0.780) is consistent with mechanisms such as blood meal diversion, physical separation via animal shelters, and temporal alignment with peak mosquito biting activity [68, 69]. This evidence strongly supports integrating livestock management into existing interventions such as insecticide-treated nets (ITNs), especially in agro-pastoral communities.

Our study demonstrates that human demographic factors are not uniform risk predictors but rather operate in a context-outcome manner. The increased biting risk associated with male-biased populations in rural settings likely reflects biological differences: males typically produce more sweat containing higher levels of volatile organic compounds [70, 71]. In rural environments, where mosquito abundance is high and activity is predominantly outdoor, these male-specific traits enhance attractiveness to foraging mosquitoes. The reversal of this effect in urban areas suggests a shift in mosquito host-seeking dynamics. Urban mosquitoes, often adapted to semi‑indoor or fragmented outdoor habitats, may be less responsive to male-specific cues, or urban males may occupy environments (e.g., better infrastructure) with reduced mosquito density, thereby offsetting their inherent biological attractiveness [72–74]. Consequently, this relationship should be interpreted cautiously: the sex ratio may serve as a proxy for direct biological or behavioral determinants of biting risk, and residual confounding or statistical artifacts cannot be ruled out.

The integrated GLMM-DLNM model demonstrated strong predictive capacity, capturing 37.8–90.8% of spatiotemporal variation, significantly outperforming climate-only models and validating the One Health approach. However, performance disparities in some urban sites revealed limitations. Monthly surveillance may obscure short-term weather effects, and unmeasured confounders such as emerging insecticide resistance evolution or microclimate variability likely contribute to residual uncertainty [26]. Furthermore, Jiangsu's advanced socioeconomic development may limit the generalizability of our findings to regions with less developed infrastructure [75, 76]. Future research should prioritize genomic surveillance of vector populations, economic evaluations of targeted interventions, and multi-country validation studies to assess the transferability of our model across different urbanization gradients.

## Conclusions

This study moves beyond documenting ecological drivers to uncovering a systematic shift in malaria vector ecology across the urban–rural gradient. We demonstrate that the One Health approach is not merely theoretical but an operational necessity for managing residual malaria transmission. Sustaining malaria elimination will require precision strategies that are adaptive to complex landscapes. In urban areas, public health efforts can leverage and enhance protective infrastructure through strategic urban planning and green space management. In rural areas, strategies must target zooprophylaxis through livestock management and mitigate the risks posed by agricultural expansion.

## Supplementary Information

Additional file 1.

## Data Availability

Data supporting the main conclusions of this study are included in the manuscript.
