# Supplementary material for: Divergent urban–rural drivers of malaria vector ecology: a 6-year One Health longitudinal study in China
Source: Parasit Vectors. 2026 Jun 15;19:360. doi: 10.1186/s13071-026-07507-w (PMC13495405; doi:10.1186/s13071-026-07507-w)
Supplement: Supplementary file 1 [file 13071_2026_7507_MOESM1_ESM.docx]

**Additional File 1**

**Table S1 to 8, Figure S1 to 2**

*Divergent urban-rural drivers of malaria vector ecology: A six-year One Health longitudinal study in China*

Table S1. Characteristics of the 12 sentinel surveillance sites.

| City | Site Name | Site Type | Key Defining Characteristic |
| --- | --- | --- | --- |
| Changzhou | Xinbei | Rural/Urban | Xinbei has a permanent population of approximately 902,900 and diverse land cover types. Besides natural land and large-scale urban-rural residential areas, it also has scattered farms for Sheep/goats, pigs, dairy Cattle/buffaloess and poultry^1^. |
| Suqian | Sihong | Rural/Urban | Sihong has a permanent population of approximately 829,900. According to the third national land survey data, its cultivated land covers 132,687.93 hectares, mainly consisting of paddy fields and dry land. The county is home to numerous farms, with pig and poultry farming being relatively common.^2^ |
| Suzhou | Zhangjiagang | Rural/Urban | Zhangjiagang has a permanent population of approximately 1,440,200 million. Its land cover is characterized by the "intertwined integration of urban industrial land and agricultural ecological land". The livestock and poultry farming in the city is mainly carried out in a standardized and intensive manner^3^. |
| Nantong | Hai'an | Rural/Urban | Hai'an has a permanent population of approximately 866,300. It is a major production area for rice and poultry eggs. The livestock and poultry farming in Haian mainly focuses on poultry and pigs^4^. |
| Xuzhou | Suining | Rural/Urban | Suining has a permanent population of approximately 1.07 million. It has vast cultivated land characterized by interspersed dry and paddy fields, and is an important grain-producing area in the Huaihai Economic Zone. Besides large-scale farms, the county also has a number of family farm-style breeding sites^5^. |
| Yancheng | Yandu | Rural/Urban | Yandu has a permanent population of approximately 612,500. Its land cover features the plain characteristics of "dense water networks and agriculture-dominated land". The core of its livestock and poultry farming is poultry (chickens, ducks) and pigs^6^. |

Table S2. One Health predictor variables. Climatic, human society (socioeconomic and urbanized), and animal hosts variables incorporated in distributed lag non-linear models of *An. sinensis* biting rate and density.

| Variables | Unit | Type | Definition | Source | Frequency |
| --- | --- | --- | --- | --- | --- |
| Climate |  |  |  |  |  |
| Temperature | °C | Continuous | The monthly mean daily mean temperature is defined as the mean of daily mean temperatures over a month, expressed in degrees Celsius. | CRU^7^ | monthly |
| Precipitation | mm | Continuous | The total amount of precipitation received in a region over the course of a month, measured in millimeters. | CRU^7^ | monthly |
| Water vapor | hPa | Continuous | This refers to the partial pressure of water vapor molecules in the atmosphere, which is the contribution of water vapor alone to the atmospheric pressure, measured in hectopascals . | CRU^7^ | monthly |
| Human society |  | Continuous |  |  |  |
| Socioeconomic |  |  |  |  |  |
| Sex ratio | the number of males per 100 females | Continuous | The sex ratio is defined as the ratio of males to females in the total population, with females standardized to a value of 100. This ratio is calculated by dividing the number (or proportion) of males by the number (or proportion) of females and then multiplying the result by 100. | Statistical Yearbook^1-6^ | annual |
| Population density | people/km² | Continuous | This indicator quantifies the population density of a specific geographic area. It is defined as the total population divided by the total area, with the result expressed in people per square kilometer . | Statistical Yearbook^1-6^ | annual |
| Rural per capita disposable income | RMB | Continuous | This refers to the income that rural residents can freely dispose of, which represents the total amount available for final consumption expenditure and savings, measured in yuan. | Statistical Yearbook^1-6^ | annual |
| Urban per capita disposable income | RMB | Continuous | This refers to the income that urban residents can freely dispose of, which represents the total amount available for final consumption expenditure and savings, measured in yuan. | Statistical Yearbook^1-6^ | annual |
| Crop cultivated area | thousand hectares | Continuous | This term refers to the total area cultivated or transplanted with crops.It encompasses all land utilized for crop production, respective of whether it is classified as arable or non-arable. Additionally, it includes regions that have been replanted or supplemented in response to disasters. The measurement unit is in thousand hectares. | Statistical Yearbook1-6 | annual |
| Urbanization |  | Continuous |  |  |  |
| Urbanization rate | % | Continuous | This variable represents the proportion of the population residing in urban areas cmpared to the total population of a region. It reflects the demographic distribution between urban and rural areas, providing insights into urbanization trends and their implications for regional development. | Statistical Yearbook1-6 | annual |
| Road area | 10,000 m² | Continuous | This refers to areas of various paved roads with a width of 3.5 meters or more, including the road surface area as well as adjents squares, bridges, and tunnels, measured in ten thousand square meters. | Statistical Yearbook1-6 | annual |
| Sewage treatment rate | % | Continuous | This variable represents the proportion of sewage that is processed by treatment facilities compared to the total volume of sewage discharged over a designated time frame. | Statistical Yearbook1-6 | annual |
| Animal hosts |  |  |  |  |  |
| Cattle/buffaloes | 10,000 heads | Continuous | Cattle and buffaloes being raised at Year-end. | Statistical Yearbook, Jiangsu Provincial Department of Agriculture1-6 8 | annual |
| Sheep/goats | 10,000 heads | Continuous | Sheep/goats and goats being raised at Year-end. | Statistical Yearbook, Jiangsu Provincial Department of Agriculture^1-6 8^ | annual |
| Hogs | 10,000 heads | Continuous | Hogs being raised at Year-end. | Statistical Yearbook, Jiangsu Provincial Department of Agriculture^1-6 8^ | annual |

Table S3. The characteristics of climatic, human society (socioeconomic and urbanized), and animal hosts variables incorporated in distributed lag non-linear models of *An. sinensis* biting rate and density.

| Variables | Uints | Mean | SD | Min | Percentile | | | Max |
| --- | --- | --- | --- | --- | --- | --- | --- | --- |
|  |  |  |  |  | 25th | 50th | 75th |  |
| Temperature | °C | 24.382 | 3.941 | 16.000 | 22.689 | 24.700 | 27.963 | 30.133 |
| Precipitation | mm | 153.818 | 98.550 | 14.440 | 72.033 | 129.783 | 223.653 | 440.560 |
| Water vapor | hPa | 23.692 | 5.899 | 10.600 | 19.400 | 23.433 | 29.644 | 31.800 |
| Sex ratio | the number of males per 100 females | 98.932 | 4.731 | 90.840 | 95.287 | 97.292 | 103.666 | 106.675 |
| Population density | people/km² | 873.532 | 414.793 | 404.000 | 584.000 | 731.000 | 1281.000 | 1757.678 |
| Rural per capita disposable income | RMB | 24469.970 | 7331.620 | 13625.000 | 18827.000 | 23354.000 | 29000.000 | 41859.000 |
| Urban per capita disposable income. | RMB | 44877.409 | 15402.502 | 22953.000 | 31018.000 | 43739.000 | 56651.000 | 77939.000 |
| Crop cultivated area | thousand hectares | 747.344 | 445.710 | 165.050 | 209.650 | 766.265 | 1178.480 | 1390.490 |
| Urbanization rate | % | 64.757 | 8.556 | 37.005 | 61.582 | 63.475 | 67.895 | 87.591 |
| Road area | 10,000 m² | 7111.974 | 4056.149 | 4126.000 | 4783.000 | 5528.080 | 6491.000 | 16925.000 |
| Sewage treatment rate | % | 94.562 | 2.918 | 87.100 | 92.900 | 95.200 | 96.600 | 100.000 |
| Cattle/ buffaloes | 10,000 heads | 3.674 | 3.498 | 0.006 | 0.890 | 2.305 | 6.350 | 14.100 |
| Sheep/goats | 10,000 heads | 149.401 | 113.856 | 3.640 | 29.540 | 140.540 | 255.870 | 382.760 |
| Hogs | 10,000 heads | 62.112 | 59.953 | 0.171 | 5.650 | 49.855 | 103.210 | 187.950 |

Table S4. A summary of climatic drivers with elevated risk on vector dynamics across habitats in the rural and urban environment.

| Site | Temperature | | Precipitation | |
| --- | --- | --- | --- | --- |
|  | IRR < 1 | IRR >1 | IRR < 1 | IRR >1 |
| **Biting rate** |  |  |  |  |
| Rural indoor | < 24.341 | (24.469, 28.053) | (154.615, 345.372) | (80.328 152.583) |
| Rural outdoor | < 21.597 | - | (154.745, 305.440) | (58.444, 152.234) |
| **Adult density** |  |  |  |  |
| Rural indoor | < 24.357 | (24.415, 29.512) | (154.630, 376.292) | (67.566, 152.546) |
| Rural outdoor | < 24.343 | (24.460, 30.133) | (154.554, 335.236) | (70.545, 152.743) |
| Urban residential | < 22.915 | (28.247, 28.383) | (154.751, 256.490) | (88.756 152.196),  > 364.830 |
| Urban parksl | < 21.059 | (28.831, 30.133) | - | > 320.399 |

Table S5. Key drivers of An. sinensis biting rates and density across habitats in the Gamma-DLMN model. Incidence rate ratios (95% CIs) were presented to show the associations between anthropogenic and zoogenic variables and vector biting rates and density. Statistically significant (p < 0.05) variables are marked by *, and those with p < 0.01 are marked by **

| Variable | Unit | Rural IRR (95%CI) | | Urban IRR (95%CI) | |
| --- | --- | --- | --- | --- | --- |
|  |  | Indoor | outdoor | Residential | Parks |
| **Adult density** |  |  |  |  |  |
| Male-biased sex ratio | 1 male per 100 females | 0.9745(0.8911-1.0657) | 1.1326(1.0199-1.2576)* | 0.8985(0.8306-0.9718)** | 0.902(0.8222-0.9896)* |
| Population density | 100 people/km2 | — | — | 0.8637(0.7123-1.0472) | 0.8154(0.647-1.0278) |
| Crop cultivated area | 10 thousand hectares | 1.0125(0.9489-1.0803) | 1.0329(1.0107-1.0557)** | — | — |
| Urbanization rate | 1% | 1.0468(1.0167-1.0779)** | 1.0392(1.0095-1.0697)** | 1.0432(1.0016-1.0865)* | 1.0744(1.0381-1.1121)** |
| Sewage treatment rate | 1% | 0.9534(0.8827-1.0297) | 0.9867(0.9206-1.0575) | 0.915(0.853-0.9816)* | 0.9681(0.8834-1.061) |
| Road area | 1 million m2 | 0.9717(0.9227-1.0232) | 1.0124(0.9928-1.0323) | 0.9925(0.9736-1.0118) | 1.0003(0.9798-1.0212) |
| Cattle/buffaloes | 10,000 heads | 1.0709(0.8436-1.3595) | 0.839(0.6931-1.0156) | — | — |
| Sheep/goats | 10,000 heads | 0.9636(0.9458-0.9817)* | 0.9944(0.9823-1.0067) | — | — |
| Hogs | 10,000 heads | 0.999(0.9961-1.0019) | 0.999(0.9958-1.0022) | — | — |
| **Biting rate** |  |  |  | — | — |
| Male-biased sex ratio | 1 male per 100 females | 1.1171(1.0426-1.197)** | 1.1778(1.0851-1.2784)** | — | — |
| Crop cultivated area | 10 thousand hectares | 1.0293(1.0183-1.0404)** | 1.0336(1.0196-1.0478)** | — | — |
| Urbanization rate | 1% | 0.9983(0.9684-1.0291) | 1.0879(1.0574-1.1192)** | — | — |
| Sewage treatment rate | 1% | 0.9069(0.8418-0.977)* | 0.929(0.8581-1.0057) | — | — |
| Road area | 1 million m2 | 0.9981(0.9933-1.0028) | 1.0001(0.9901-1.0102) | — | — |
| Cattle/buffaloes | 10,000 heads | 0.7064(0.6342-0.7868)** | 0.7209(0.6219-0.8356)** | — | — |
| Sheep/goats | 10,000 heads | 0.991(0.9881-0.9938)** | 1.0002(0.9934-1.007) | — | — |
| Hogs | 10,000 heads | 1.0006(0.9979-1.0033) | 0.9978(0.9948-1.0007) | — | — |

Table S6. Fit statistics for the Gamma-DLMN model.

| Model | Marginal R^2^ | Conditional R^2^ | AIC |
| --- | --- | --- | --- |
| Rural indoor biting rate | 0.758 | 0.758 | 863.6 |
| Rural outdoor biting rate | 0.619 | 0.704 | 991.5 |
| Rural indoor density | 0.424 | 0.958 | 1969.6 |
| Rural outdoor density | 0.640 | 0.832 | 1963.6 |
| Urban residential density | 0.555 | 0.778 | 929.5 |
| Urban park density | 0.456 | 0.723 | 1007.5 |

Table S7. The importance of One Health factors in the biting rate and mosquito density of Random Forests Models and the model results.

| **Variables** | | **%IncMSE** | **IncNodePurity** | **Mse** | **Adjusted R^2^** |
| --- | --- | --- | --- | --- | --- |
| **Biting rate** | | | |  |  |
| **Rural indoor** | Temperature | 33.262 | 605.102 | 0.631 | 0.821 |
|  | Crop cultivated area | 15.979 | 140.317 |  |  |
|  | Male-biased sex ratio | 13.356 | 204.073 |  |  |
|  | Cattle/buffaloes | 11.537 | 79.592 |  |  |
|  | Sheep/goats | 33.262 | 605.102 |  |  |
|  | Precipitation | 15.979 | 140.317 |  |  |
| **Rural outdoor** | Temperature | 36.786 | 587.194 | 0.634 | 0.832 |
|  | Crop cultivated area | 25.470 | 215.588 |  |  |
|  | Male-biased sex ratio | 18.946 | 262.380 |  |  |
|  | Cattle/buffaloes | 16.830 | 128.237 |  |  |
|  | Precipitation | 5.694 | 134.043 |  |  |
| **Density** | | | |  |  |
| **Rural indoor** | Temperature | 26.375 | 1107154.920 | 1325.360 | 0.763 |
|  | Urbanization rate | 4.771 | 339005.552 |  |  |
|  | Precipitation | 2.903 | 236689.647 |  |  |
|  | Sheep/goats | 0.125 | 116026.346 |  |  |
| **Rural outdoor** | Temperature | 41.810 | 2541827.290 | 1800.498 | 0.806 |
|  | Precipitation | 9.362 | 453746.118 |  |  |
|  | Crop cultivated area | 6.334 | 338031.743 |  |  |
| **Urban residential** | Temperature | 41.997 | 1068.272 | 1.008 | 0.838 |
|  | Precipitation | 19.844 | 527.027 |  |  |
|  | Male-biased sex ratio | 13.224 | 248.274 |  |  |
|  | Urbanization rate | 12.885 | 217.115 |  |  |
|  | Population density | 12.652 | 168.840 |  |  |
| **Urban parks** | Temperature | 40.672 | 4539.444 | 3.790 | 0.837 |
|  | Precipitation | 13.339 | 1754.477 |  |  |
|  | Male-biased sex ratio | 12.416 | 803.111 |  |  |
|  | Urbanization rate | 10.401 | 643.628 |  |  |
|  | Population density | 10.040 | 629.945 |  |  |

Table S8. Correlation between model predicted values and observed values at different locations

| Correlation | | Xinbei | Sihong | Zhangjiagang | Haian | Suining | Yandu |
| --- | --- | --- | --- | --- | --- | --- | --- |
| Rural indoor biting rate | *r* | 0.794 | 0.481 | 0.558 | 0.642 | 0.236 | 0.789 |
|  | *P* | < 0.001 | 0.005 | 0.001 | < 0.001 | 0.193 | < 0.001 |
| Rural outdoor biting rate | *r* | 0.345 | 0.566 | 0.802 | 0.551 | 0.480 | 0.664 |
|  | *P* | 0.058 | 0.001 | < 0.001 | 0.001 | 0.005 | < 0.001 |
| Rural indoor density | *r* | 0.856 | 0.816 | 0.600 | 0.510 | 0.685 | 0.706 |
|  | *P* | < 0.001 | < 0.001 | < 0.001 | 0.003 | < 0.001 | < 0.001 |
| Rural outdoor density | *r* | 0.543 | 0.859 | 0.799 | 0.781 | 0.207 | 0.596 |
|  | *P* | 0.002 | < 0.001 | < 0.001 | < 0.001 | 0.256 | < 0.001 |
| Urban residential density | *r* | 0.103 | 0.675 | 0.481 | 0.908 | 0.501 | 0.728 |
|  | *P* | 0.580 | < 0.001 | 0.005 | < 0.001 | 0.003 | < 0.001 |
| Urban park density | *r* | 0.133 | 0.576 | 0.378 | 0.877 | 0.718 | 0.601 |
|  | *P* | 0.476 | 0.001 | 0.033 | < 0.001 | < 0.001 | < 0.001 |

Figure S1. Monthly based temporal dynamics of *An. sinensis* populations in April to October. Monthly trends in A, rural biting rates and B rural density and urban density.

Figure S2. Distinct exposure-lag-response relationships of climate drivers between rural and urban habitats in in Gamma-DLNM model model.

References.

1	Changzhou Municipal Statistics Bureau. Statistical Yearbook. 2024. Available: https://tjj.changzhou.gov.cn/class/MFCMMFBD [Accessed 8 May 2025].

2	People's Government of Suqian Municipality. Statistical Yearbook. 2025. Available: https://suqian.gov.cn/cnsq/xxgktjnj/xxgk_list.shtml [Accessed 8 May 2025].

3	People's Government of Suzhou Municipality. Statistical Yearbook. 2024. Available: https://www.suzhou.gov.cn/szsrmzf/tjnj/xxgkzdly.shtml [Accessed 8 May 2025].

4	Nantong Municipal Statistics Bureau. Statistical Yearbook. 2024. Available: https://tjj.nantong.gov.cn/ntstj/tjnj/tjnj.html [Accessed 8 May 2025].

5	Xuzhou Municipal Statistics Bureau. Annual Data. 2025. Available: https://tj.xz.gov.cn/dynamic/zwgk/govInfoPubright.html?categorynum=003016001 [Accessed 8 May 2025].

6	Yanchen Municipal Statistics Bureau. Statistical Yearbook. 2024. Available: https://tjj.yancheng.gov.cn/col/col1779/index.html [Accessed 8 May 2025].

7	Climate Resesrch Unit. CRU TS Version 4.06. 2024. Available: https://crudata.uea.ac.uk/cru/data/hrg/cru_ts_4.06/ [Accessed 8 May 2024].

8	Jiangsu Provincial Department of Agriculture and Rural Affairs. Annual Statistical Data. 2025. Available: https://nynct.jiangsu.gov.cn/col/col12552/index.html?number=18 [Accessed 8 May 2025].
